# Framework for Approaching Patients with Suboptimal Deep Brain Stimulation Therapy Response despite Stimulation Optimization

**DOI:** 10.1002/mdc3.70609

**Published:** 2026-03-27

**Authors:** Pratik Talati, Meredith Spindler, Casey H. Halpern

**Affiliations:** ^1^ Department of Neurosurgery, Penn State Health College of Medicine Hershey Medical Center Hershey Pennsylvania USA; ^2^ Department of Neurosurgery, Perelman School of Medicine University of Pennsylvania Philadelphia Pennsylvania USA; ^3^ Department of Neurology Perelman School of Medicine of the University of Pennsylvania Philadelphia Pennsylvania USA

**Keywords:** deep brain stimulation, DBS, DBS rescue clinic, rescue DBS

Deep brain stimulation (DBS) is a widely used surgical treatment for movement disorders. While over 90% of patients report meaningful symptom improvement after DBS implantation, rare cases of dissatisfaction exist.^1^ This letter proposes a framework for managing DBS patients who have already undergone programming optimization by a DBS specialist. Such patients can benefit from a DBS rescue clinic that leverages a multidisciplinary team involving movement‐disorder neurologists and functional neurosurgeons to provide advanced troubleshooting and revision strategies to improve stimulation‐modifiable symptoms of an existing DBS device.

The first and most important step in evaluating a dissatisfied DBS patient is to confirm the surgical indication (ie, DBS‐responsive symptoms and diagnosis). This involves a thorough patient interview, focusing on history‐taking and symptom presentation, medication trial and response, and understanding the most troublesome symptoms and goal of surgery. Clinical records, including on–off levadopa testing, on–off stimulation testing, monopolar review, and stimulation trials should be obtained as well as pre‐operative and post‐operative imaging to facilitate DBS lead reconstruction prior to the visit. Reviewing the on–off levadopa testing results can determine whether the bothersome symptoms are levodopa‐responsive, and thus more likely to be responsive to DBS. During the clinical visit, impedance testing can be completed to verify hardware integrity. It is also possible new symptoms have developed since implantation that may not be optimally managed with the present DBS system, which may merit another evaluation on and off medications and on and off stimulation.

If the patient's most bothersome symptoms are those typically responsive to DBS, the next step is to assess suitability for intracranial lead revision or implantation of additional rescue leads to a different intracranial target. Certain patients may be unsuitable for intracranial surgery due to medical comorbidities limiting tolerance of another surgery, coagulopathy or inability to hold anticoagulation, or neuropsychiatric contraindications. For patients who are not candidates for intracranial surgery but continue to experience stimulation‐induced side effects, updating the implantable pulse generator may allow for greater stimulation optimization. Modern generators can provide access to newer features such as anodic blocking, multiple independent current control, pulse width adjustment, and adaptive stimulation to help manage stimulation‐induced symptoms.^2^, ^3^ Alternative strategies exist if the stimulation is not causing side effects but is providing insufficient benefit, including lesional procedures. These procedures can include stereotactic pallidotomy, thalamotomy, or subthalamotomy; MR guided focused ultrasound; or radiofrequency ablation.^4^

For patients who are candidates for intracranial lead revision or implantation, the next step is to perform a DBS lead localization, which ideally combines the patient's pre‐ and post‐operative imaging to assess the intracranial lead placement. Lead localization provides a detailed understanding of the implanted leads’ position relative to other brain structures, helping to identify reasons for reduced therapeutic effectiveness. If bothersome stimulation‐induced side effects are present despite optimal programming, a trial off stimulation is warranted to determine whether the stimulation is providing benefit for underlying symptoms. Well‐placed leads providing benefit but with bothersome symptoms that are typically responsive to DBS should have already undergone a trial of advanced programming using directional steering, interleaving stimulation, anodal blocking, current cycling, local field potential‐based adaptive stimulation, and/or imaging‐based automated current optimization as determined by device capabilities prior to the clinic evaluation. The outcome can guide multidisciplinary discussions about whether to explore lead revision (to directional leads with current steering capabilities),^5^ additional rescue leads to another target, lesional procedures, or no intervention. Conversely, if the leads are in a suboptimal position, the DBS system should be removed followed by implantation of a new system into the most appropriate target guided by high quality imaging. While there may be some variations in practice, an updated pre‐operative MRI and CT scan is preferred after DBS removal.

Decisions regarding surgical revisions in patients dissatisfied with DBS should ideally be made by a multidisciplinary team of movement disorders specialists, functional neurosurgeons, and other potentially relevant specialists such as neuropsychologists and psychiatrists. Our proposed, detailed framework is illustrated in Fig. 1. This decision tree is derived from our robust experience and is intended to serve as a guide for the multidisciplinary team to assist in the complicated management of patients dissatisfied with DBS therapy in achieving better outcomes.

**Figure 1:**
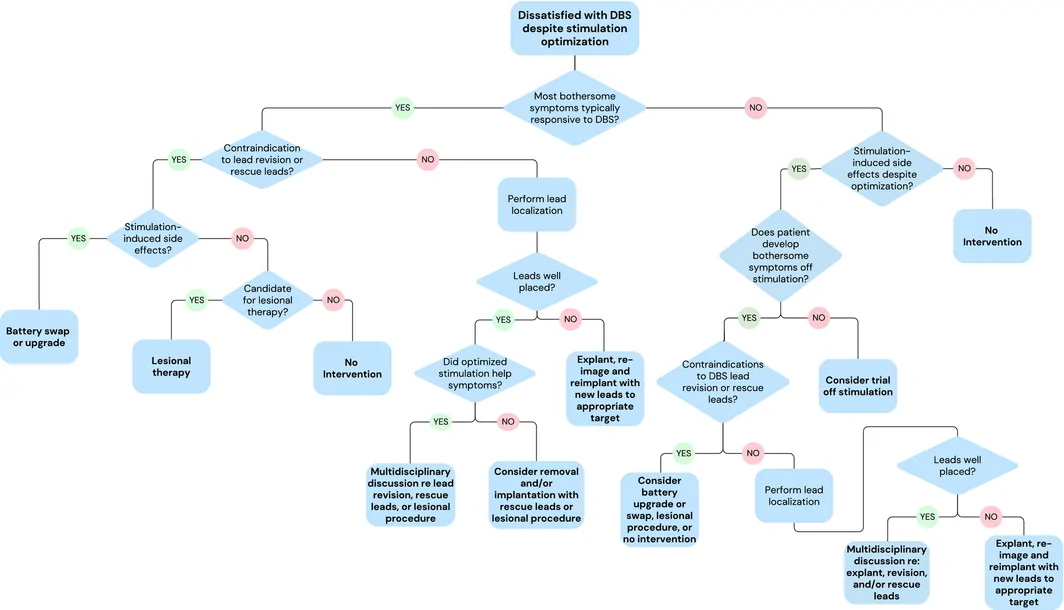
Proposed flowchart for a rescue clinic workflow in patients who are dissatisfied with DBS therapy despite stimulation optimization. Battery upgrade denotes replacing an implanted pulse generator with a newer model from the *same manufacturer* to allow for enhanced programming capabilities. Battery swap denotes an implanted pulse generator replacement to a *different manufacturer* to access additional programming features not available in the original system. Lesional procedure denotes focused ultrasound, stereotactic pallidotomy/thalamotomy/subthalamotomy, or radiofrequency ablation. Rescue leads denote additional intracranial lead placement in addition to the existing intracranial leads.

The decision to proceed with an additional surgery to optimize DBS response requires extensive case review and careful, comprehensive discussion of options with patients who may understandably be reluctant due to their dissatisfaction with the initial procedure or may have elevated surgical risk due to progression of disease and prior surgical intervention. While not all centers may have the resources to follow such a time and resource‐intensive framework for dissatisfied patients, the growing number of centers performing DBS as well as the expanding use of DBS‐specialized advanced practice providers may facilitate the development of such rescue clinics. Notably, we have found that most patients assessed with our framework are surgical candidates and undergo intracranial lead revision, addition of intracranial leads, or a pulse generator change (to the same or different manufacturer), highlighting the value of a rescue clinic.

## Author Roles

(1) Research project: A. Conception, B. Organization, C. Execution; (2) Manuscript Preparation: A. Writing of the first draft, B. Review and Critique.

P.T.: 1A, 1B, 1C, 2A.

M.S.: 1B, 2B.

C.H.H.: 1A, 1B, 2B.

## Disclosures

**Ethical Compliance Statement:** The authors confirm that the approval of an institutional review board was not required for this work. Informed consent was not needed for this study. We confirm that we have read the Journal's position on issues involved in ethical publication and affirm that this work is consistent with those guidelines.

**Funding Sources and Conflict of Interest:** No specific funding was received for this work; MS and CH have received honoraria from Boston Scientific and Medtronic but have no conflict of interest relevant to this work; PT has no conflict of interest relevant to this work.

**Financial Disclosures for the previous 12 months:** CH has funding support from the following grants: NIH DP1OD040905‐0, NIH UH3 NS113769, R01 MH124760, UH3 NS103446. MS and PT have no disclosures to report.

## Financial Disclosures and Conflicts of Interest

Author disclosures are available in the Supporting Information.

## Supporting information


**Data S1.** Supporting Information.

## Data Availability

Data sharing not applicable to this article as no datasets were generated or analysed during the current study.
